# Flow Cytometric Quantification of All Phases of the Cell Cycle and Apoptosis in a Two-Color Fluorescence Plot

**DOI:** 10.1371/journal.pone.0068425

**Published:** 2013-07-30

**Authors:** Christine Vignon, Christelle Debeissat, Marie-Thérèse Georget, Didier Bouscary, Emmanuel Gyan, Philippe Rosset, Olivier Herault

**Affiliations:** 1 Université François-Rabelais de Tours, Tours, France; 2 CNRS, UMR 7292, LNOx team, Tours, France; 3 CHRU de Tours, Service d’Hématologie Biologique, Tours, France; 4 Institut Cochin, Université Paris Descartes, CNRS UMR 8104, Paris, France; 5 CHRU de Tours, Service d’Hématologie Clinique et Thérapie Cellulaire, Tours, France; 6 CHRU de Tours, Service de Chirurgie Orthopédique et Traumatologique, Tours, France; French Blood Institute, France

## Abstract

An optimal technology for cell cycle analysis would allow the concomitant measurement of apoptosis, G0, G1, S, G2 and M phases in combination with cell surface phenotyping. We have developed an easy method in flow cytometry allowing this discrimination in an only two-color fluorescent plot. It is based on the concomitant use of 7-amino-actinomycin D and the antibodies anti-Ki67 and anti-phospho(Ser10)-histone H3, both conjugated to Alexa Fluor®488 to discriminate G0 and M phases, respectively. The method is particularly valuable in a clinical setting as verified in our laboratory by analyzing human leukemic cells from marrow samples or after exposure to cell cycle modifiers.

## Introduction

Considering the diagnostic importance of a detailed cell cycle analysis in a wide variety of diseases and their therapeutic management, such as evaluation the quiescent status evaluation of stem cells and the monitoring of the mitotic index in antimitotic treatments, a relevant interest exists for the development of methods for simultaneously detecting the apoptosis and all phases of the cell cycle, including the distinction of the G0 and M phases. The flow cytometric approach described in this protocol is a useful technology for studying concomitantly all these parameters in a heterogeneous cell population. This method identifies quiescent cells by binding the monoclonal antibody anti-Ki-67 to a nuclear antigen present in all cells that are in the G1, S, G2, and M phases of the cell cycle, but not those in the G0 phase [1]. Moreover, the cells engaged in mitosis are identified by staining the histone H3 phosphorylated at serine 10 [2]. The other cell cycle phases and the apoptotic state are classically quantified by double-strand DNA staining with 7-amino-actinomycin D (7-AAD) [3].

The Ki-67 antigen is expressed in the nucleus of dividing cells and is not during G0 phase. During interphase, it is associated with nucleolar components, and it is on the surface of the chromosomes during M phase. Because of the strict association of Ki-67 expression with cell proliferation, anti-Ki-67 antibodies are useful for the flow cytometric identification, quantification, and monitoring of cell populations in the G0 phase [1], [4], [5]. In eukaryotes, modulation of chromatin structure has an important role in the regulation of transcription. The nucleosome is the primary building block of chromatin [6] and the amino-terminal tails of core histones undergo various post-translational modifications, including phosphorylation [7], [8]. Phosphorylation at Ser10 of histone H3 is strongly correlated with chromosome condensation during mitosis [2] and anti-phosphorylated (ser10) H3 is useful for the flow cytometric identification, quantification and monitoring of cell populations in the M phase [9].

We document here the successful utilization of a method of discriminating concomitantly apoptosis and the phases of the cell cycle in a model of leukemic cells exposed to inducers of cell cycle perturbations. The value of this method to analyze heterogeneous cell populations is shown using a mix of B and T cells and using marrow cells from acute myeloid leukemia (AML).

## Materials and Methods

### Cells

The human cell lines, KG1a (acute myelogenous leukemia), Jurkat (T cell leukemia) and Raji (Burkitt’s B cell chronic lymphoma) were obtained from HPA Culture Collections (Salisbury, UK) and MV4–11 (acute myelomonocytic leukemia) from the German Resource Centre for Biological Material (Braunschweig, Germany). KG1a and MV4–11 cells were cultured in MEM alpha medium (Life Technologies, Villebon-sur-Yvette, France) supplemented with 10% heat-inactivated fetal bovine serum (FBS) (Life Technologies), 2 mM L-glutamine (Life Technologies), 100 units/mL penicillin and 100 µg/mL streptomycin (Boehringer-Mannheim, Mannheim, Germany). For the Jurkat and Raji cells, MEM alpha medium was replaced by RPMI 1640 (Fisher Scientific, Illkirch, France). Bone marrow (BM) and peripheral blood cells were collected from healthy donors and patients who had provided a signed written consent. These samplings were performed according to the ethical rules of our country and approved by our local ethic committee named “Comité de Protection de la Personne (CPP)-Tours Ouest 1”. BM leukemic cells were obtained from patients with diagnosed AML (Department of Clinical Hematology, University Hospital, Tours, France). Normal BM culture-amplified mesenchymal stromal/stem cells (MSCs) were produced from BM cells of patients undergoing orthopaedic surgery (Department of Orthopedic Surgery, University Hospital, Tours, France). Cells were centrifuged, seeded in flasks at a density of 5×10^3^ per cm^2^ in MEM alpha culture medium supplemented with 10% FCS, 2 mM L-glutamine, 100 µg/mL of penicillin G and incubated at 37°C in 95% humidified air and 5% CO_2_.

### Induction of cell cycle perturbations

The inhibition of mitosis and the induction of apoptosis in KG1a and MV4–11 cells were induced respectively by exposure to camptothecin (Sigma-Aldrich, Saint-Quentin Fallavier, France), a cytotoxic quinoline alkaloid which inhibits the DNA enzyme topoisomerase I [10], [11] and by AZD8055 (AstraZeneca Cancer & Infection Research Area, Alderley Park, UK) [12], a selective inhibitor of mTOR kinase, respectively. Cells were seeded at 2×10^5^ cells/mL (5% CO_2_ incubator at 37°C). KG1a cells were cultured for 6h with camptothecin at a final concentration of 1 µM and MV4–11 cells were cultured for 24 h with AZD8055 at a final concentration of 10 nM and 100 nM. The stock solutions were diluted to ensure a final concentration of <0.03% for DMSO (Sigma-Aldrich). Control cultures were treated with an equivalent volume of DMSO in MEM alpha medium which did not induce apoptosis.

Quiescence was induced in KG1a cells by contact with BM MSCs [13]. Adherent culture-amplified MSCs were used at passage 2 (P2). KG1a cells were co-cultured on P2-MSCs for 72 h (37°C in 95% humidified air and 5% CO_2_) at a starting concentration of 1.5×10^4^/cm^2^.

The accumulation of KG1a cells in the M phase was induced by exposure to colcemid (KaryoMax Colcemid, Life Technologies), used for arresting the dividing cell at metaphase of mitosis. Cells were cultured 30 min and 1 h with colcemid at a final concentration of 0.1 µg/mL.

Lymphocytes stimulation was induced by exposure to phytohemagglutinin (PHA) (Remel™, Oxoid™, Haarlem, The Netherlands), which is used to stimulate mitotic division of lymphocytes. Whole blood cells were cultured 72 h with PHA at a final concentration of 170 µg/mL according to the manufacturer’s recommandations.

All experiments were performed in triplicate.

### Cell Cycle Staining

The lysis of red blood cells from BM or peripheral blood samples was induced by the addition of 1 mL of BM and 20 mL of ammonium chloride (10 min at 37°C and spun at 500 *g* for 5 min). Before staining, the cells were washed twice by centrifugation in phosphate-buffered saline (PBS) at 500 *g* for 5 min. Then, 10^6^ cells were permeabilized with 1 mL of ice cold ethanol (1 h, 4°C). Following two washes with PBS, 1% FBS and 0.25% Triton X-100 (PFT), the cells were stained in 200 µL of PFT for 30 min at room temperature in the dark with 10 µg 7-AAD (Sigma-Aldrich), 5 µL Alexa Fluor®488-conjugated anti-human Ki67 mAb (B56) (Becton-Dickinson, Pont-de-Claix, France) and 3 µL Alexa Fluor®488-conjugated anti-phospho(ser10)-histone H3 polyclonal antibody (Cell Signaling Technology, Danvers, MA). A control tube was prepared with 10 µg 7-AAD and 5 µL of Alexa Fluor®488-conjugated mouse IgG1 (Becton-Dickinson). After 2 washes with PFT, the cells were stained with 10 µL of APC-Cy7-conjugated anti-CD45 (A20) or with 5 µL of Horizon™ V450-conjugated anti-CD3 (UCHT1) antibodies from Becton-Dickinson, followed by incubation for 20 min at 4°C. Cells were then washed twice with PBS, centrifuged for 5 min at 500 g and re-suspended in 300 µL of PBS. Samples were analyzed on a FACSCanto II flow cytometer (Becton-Dickinson) equipped with three lasers, a blue (488-nm, air-cooled, 20-mW solid state), a red (633-nm, 17-mW HeNe) and a violet (405-nm, 30-mW solid state). The green fluorescence (Alexa Fluor®488 emission) was collected after passing through a 530/30 nm band pass (BP) filter. APC-Cy7 emission was detected by filtration through a 780/60 nm BP filter and Horizon™ V450 emission by filtration through a 450/50 nm BP filter. 7-AAD emission was collected after passing through a 650 nm long pass filter.

## Results and Discussion

This flow cytometric method allows a precise analysis of the impact of various functional modulators on the cell cycle. It was validated by performing three sets of experiments on hematologic cells: (1) the induction of apoptosis and cell cycle arrest by exposure to camptothecin and AZD8055, (2) the induction of quiescence by contact with primary bone marrow MSCs and (3) the promotion of cell cycle and accumulation of cells at the M-phase by treatment respectively with PHA and colcemid.

**Figure 1:**
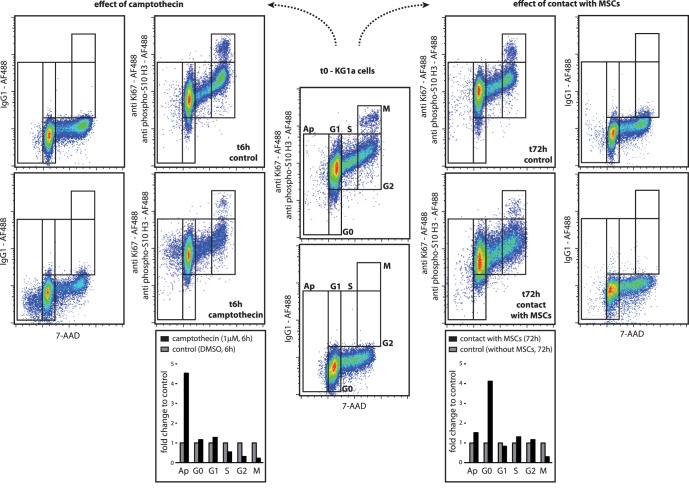
Cell cycle analysis of human KG1a leukemic cell line exposed to cell cycle modifiers. The cells were stained with 7-amino-actinomycin D (7-AAD) and antibodies anti-Ki67 and anti-phospho(Ser10)-histone H3 conjugated to Alexa Fluor®488. An isotype control staining with Alexa Fluor®488 mouse IgG1 was performed. (Left) Effects of camptothecin (1 µM, 6 h, 37°C, 5% CO_2_). The histograms present the proportions of apoptosis and all cell cycle phases normalized to those of the untreated leukemic cells. (Right) Effects of contact with bone marrow primary mesenchymal stromal/stem cells (MSCs) (72 h, 37°C, 5% CO_2_). The leukemic cells were identified by concomitant staining with APC-Cy7-conjugated anti-CD45 mAb. The histograms present the proportions of apoptosis and all cell cycle phases normalized to those of the leukemic cells without MSCs. (representative experiment).

**Figure 2:**
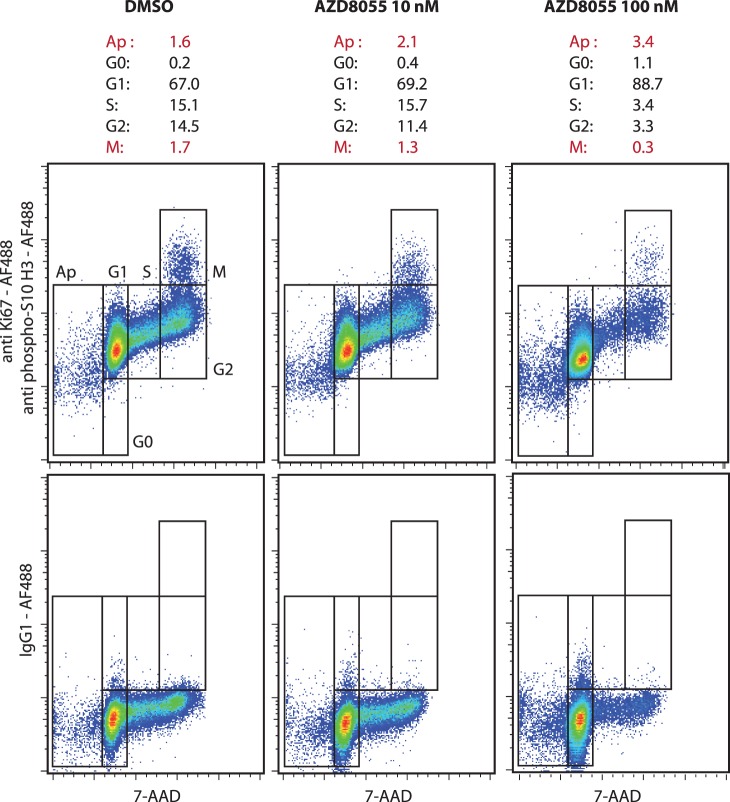
Cell cycle analysis of human MV4–11 leukemic cell line exposed to AZD8055. The cells were stained with 7-AAD and antibodies anti-Ki67 and anti-phospho(Ser10)-histone H3 conjugated to Alexa Fluor®488. An isotype control staining with Alexa Fluor®488 mouse IgG1 was performed. Effects of AZD8055 (10 nM and 100 nM, 24 h, 37°C, 5% CO_2_). The results present the percentage of cells in the apoptosis and all cell cycle phases. (representative experiment).

**Figure 3:**
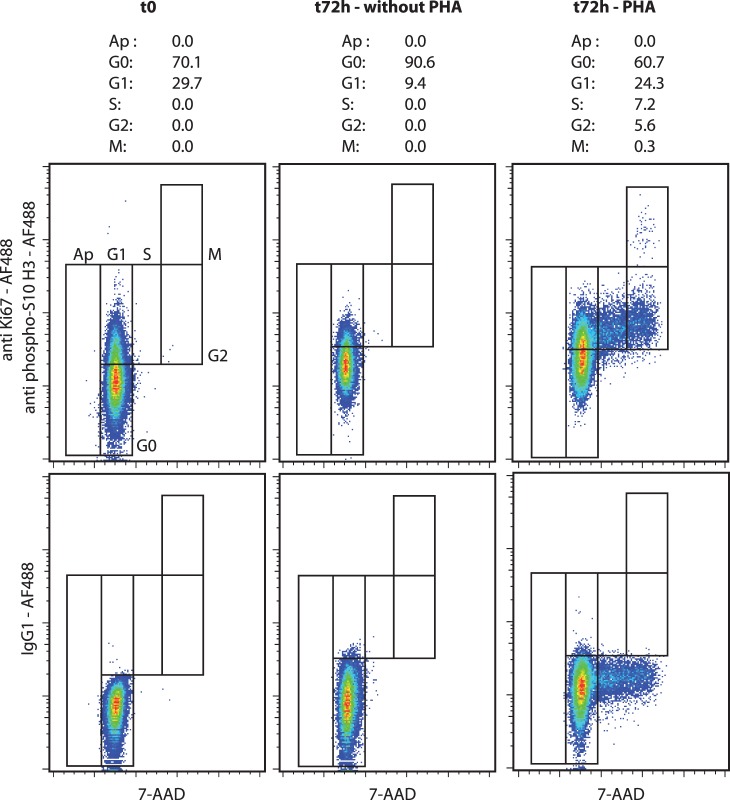
Cell cycle analysis of human lymphocytes exposed to PHA. The cells were stained with 7-AAD and antibodies anti-Ki67 and anti-phospho(Ser10)-histone H3 conjugated to Alexa Fluor®488. An isotype control staining with Alexa Fluor®488 mouse IgG1 was performed. Effects of PHA (170 µg/mL, 72 h, 37°C, 5% CO_2_). The results present the percentage of cells in the apoptosis and all cell cycle phases. (representative experiment).

**Figure 4:**
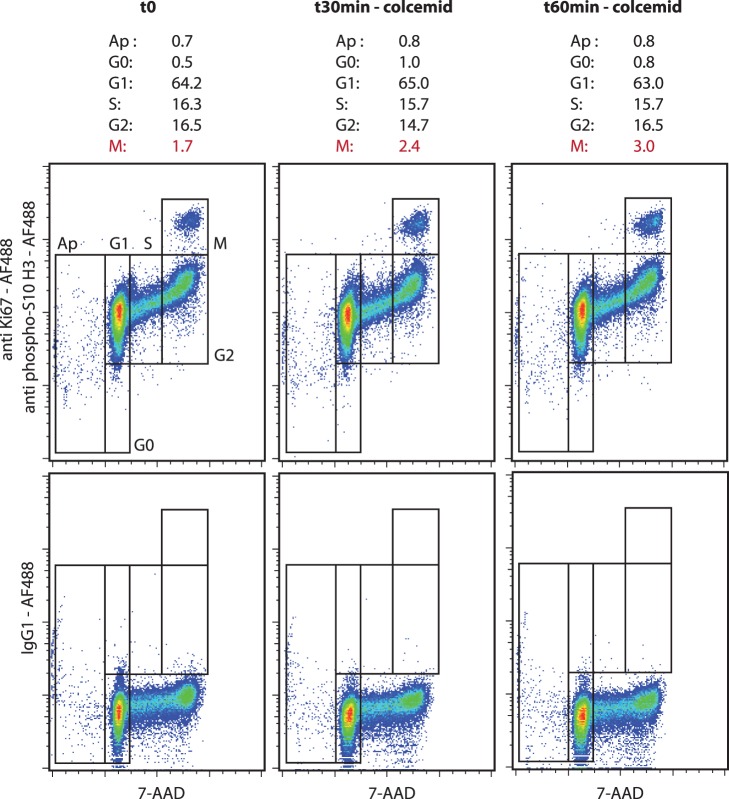
Cell cycle analysis of human KG1a leukemic cell line exposed to colcemid. The cells were stained with 7-AAD and antibodies anti-Ki67 and anti-phospho(Ser10)-histone H3 conjugated to Alexa Fluor®488. An isotype control staining with Alexa Fluor®488 mouse IgG1 was performed. Effects of colcemid (0.1 µg/mL, 30 min and 1 h, 37°C, 5% CO_2_). The results present the percentage of cells in the apoptosis and all cell cycle phases. (representative experiment).

Apoptosis in KG1a cells was induced by camptothecin (Fig. 1, left). The cell cycle characteristics of untreated KG1a cells were quantified as follows: sub-G1 0.4%, G0 0.8%, G1 67.9%, S 14.9%, G2 14.2% and M 1.8% phase. We verified the pro-apoptotic and anti-proliferative effects of exposure to camptothecin for 6 h, which induced a decrease in S, G2 and M phases (6.1%, 6.2% and 0.4%, respectively) and an increase in sub-G1 phase (1.7%). The induction of apoptosis and the inhibition of mitosis was also observed in MV4–11 cells exposed for 24 h to AZD8055 at two final concentrations (10 nM and 100 nM) both of which induced a decrease in the S, G2 and M phases and an increase in the sub-G1 phase (Fig. 2). The pro-quiescent effects of contact with bone marrow primary MSCs on the KG1a leukemic cell line was verified in co-culture experiments. As shown in Fig. 1 (right), the contact with marrow MSCs during 72 h induced an increase in the G0 phase and a decrease in the M phase (5.3% and 0.4%, respectively). This method was also tested on peripheral blood lymphocytes stimulated for 72 h with PHA (Fig. 3). As expected, the peripheral lymphocytes were mostly in a quiescent state (G0 90.6%, G1 9.4%) and PHA induced cell cycle entry with an increase in the G1, S, G2 and M phases (G0 60.7%, G1 24.3%, S 7.2%, G2 5.6%, M 0.3%). Moreover, we exposed KG1a cells to colcemid to verify the efficiency of the M phase discrimination of our method (Fig. 4). As expected, the exposure to colcemid for 30 and 60 min promoted the accumulation of cells in the M phase (2.4% and 3.0%, respectively) in comparison with untreated cells (M 1.7%).

**Figure 5:**
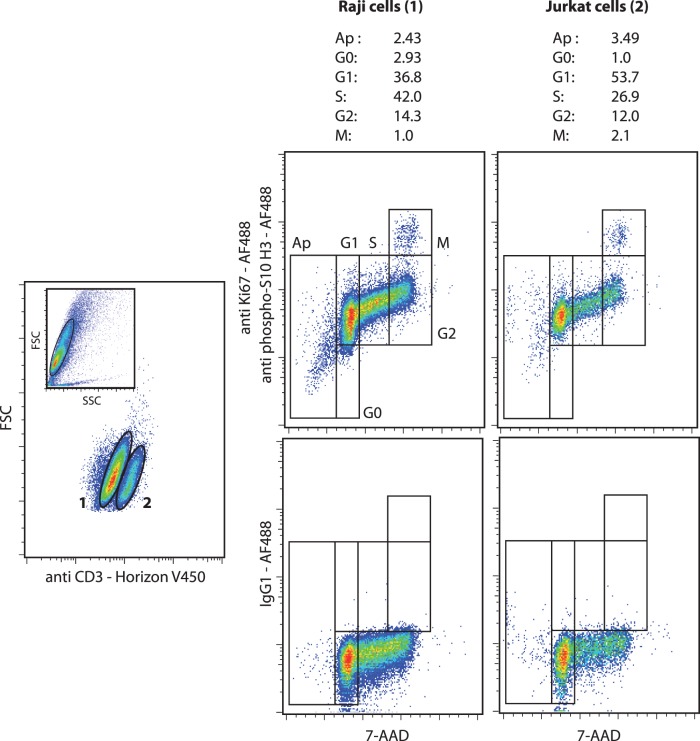
Cell cycle analysis cell subpopulations in mixed B and T cell suspension (70% Raji and 30% Jurkat cells). The cells were stained with 7-AAD, Alexa Fluor®488-conjugated anti-Ki67, Alexa Fluor®488-conjugated anti-phospho(Ser10)-histone H3 and Horizon™ V450-conjugated anti-CD3 antibodies. An isotype control staining with Alexa Fluor®488 mouse IgG1 was performed. Lymphocytes were identified by CD3/FSC gating. (representative experiment).

**Figure 6:**
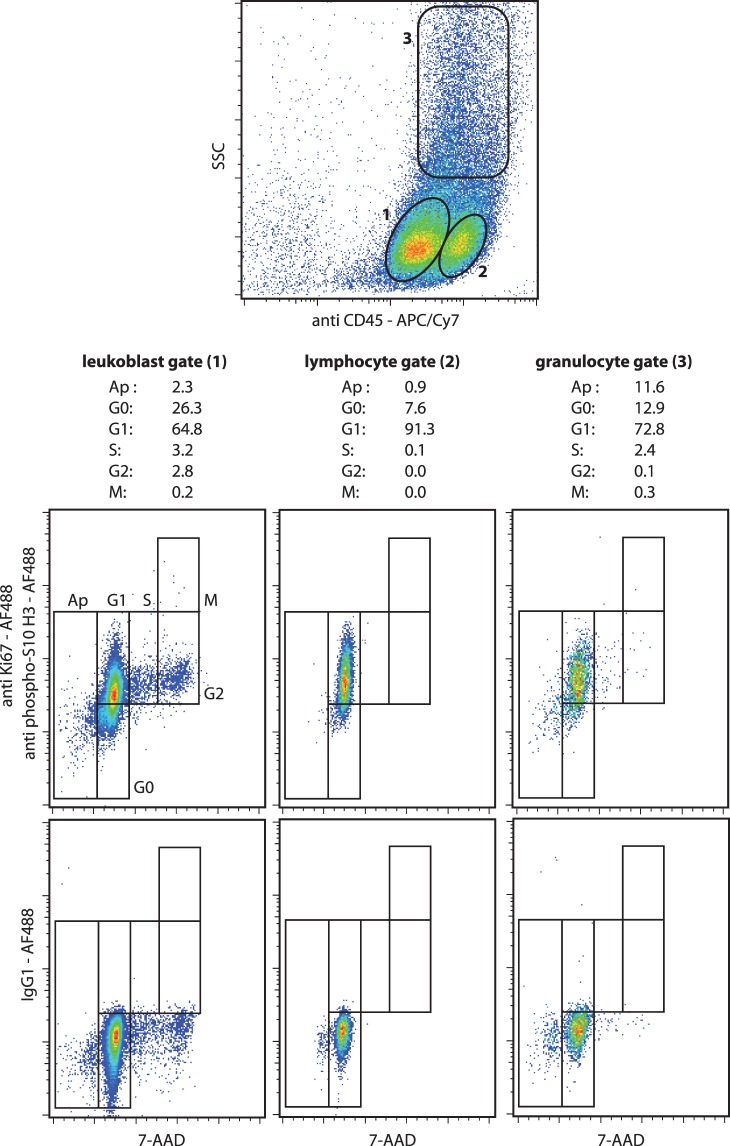
Cell cycle analysis of bone marrow cells of patient suffering from AML. The cells were stained with 7-AAD, Alexa Fluor®488-conjugated anti-Ki67, Alexa Fluor®488-conjugated anti-phospho(Ser10)-histone H3 and APC-Cy7-conjugated anti-CD45 antibodies. An isotype control staining with Alexa Fluor®488 mouse IgG1 was performed. The leukoblasts, lymphocytes and granulocytic-lineage cells were identified by CD45/SSC gating. (representative analysis).

It is interesting to note that this staining protocol preserves the membrane integrity well enough to allow the analysis of heterogeneous cell populations as shown in Fig. 5 and Fig. 6. As a first step, we mixed B and T cells (Raji 70% and Jurkat 30%, respectively) and the sub-populations were discriminated on the basis of CD3 expression. The staining of CD3 clearly identifed the two cell lines and allowed the concurrent quantification of all the cell cycle phases of each cell population (Fig. 5). In a second evaluation, the method was applied to bone marrow cells from a patient suffering from AML. The leukoblasts were identified by CD45/SSC gating [14] and as expected, leukoblasts were actively cycling conversely to lymphocytes and granulocytic-lineage cells (Fig. 6). The percentage of cells in the G0 phase in the leukoblast gate was probably overestimated due to the presence of normal hematopoietic progenitors in this SSC^low^CD45^int^ gate.

This method permits the discrimination of apoptosis and all cell cycle phases with only two fluorochromes (7-AAD and Alexa Fluor®488), providing an instantaneous photographic image of the cell cycle in a single graph with the possibility of adding other fluorochromes for the analysis of cellular sub-populations. Indeed, on one hand 7-AAD is a G-C base-specific DNA intercalator [15] which identifies the sub G1 hypoploid apoptotic cells, and the G0/G1 (2N peak), S and G2/M (4N peak) phases [3]. 7-AAD emits in the far red range of the spectrum. The emission spectra of 7-AAD (647 nm) can therefore be separated from the emissions of Alexa Fluor®488 (519 nm). On the other hand, Ki-67 antigen expression and phosphorylation at serine 10 of histone H3 can be studied concomitantly with the same fluorochrome since Ki-67 protein is absent from G0 phase cells (2N) [1] and phosphorylation at serine 10 of H3 is restricted to proliferative cells engaged in the M phase (4N) [2]. Therefore, this method allows the simultaneous discrimination of cells in a quiescent state (G0 phase), and mitotic cells (M phase) from cells in the S and G2 phases and apoptotic cells.

The method was validated on hematological cells by inducing changes in the cell cycle. It detected the expected pro-apoptotic and antiproliferative effects of camptothecin and AZD8055 exposure on leukemic cells [10], [11], [12]; we observed a decrease of leukemic cells in the S, G2 and M phases and an increase in sub-G1 phase. Moreover, the pro-quiescent effects of contact with bone marrow primary MSCs were detected by an increase of leukemic cells in the G0 phase concomitant with a decrease of cells in the M phase [13], [16]. Finally, in agreement with well-established biological effects [17], [18], the promotion of the cell cycle and the accumulation of cells at the M phase were clearly identified in the experiments with PHA and colcemid. Interestingly, as shown in Fig. 5 and Fig. 6, the permeabilization process of the protocol didn’t impair the labeling of surface antigens and allowed the analysis of heterogeneous cell populations even if ethanol is not the best fixative to preserve the membrane antigens.

In conclusion, the quick and easy analytical method we present in this article is particularly valuable in a clinical setting because it allows flow cytometric quantification and monitoring of apoptosis and concurrent analysis of all phases of the cell cycle in heterogenous cell populations.
